# Wie alles begann: die Entwicklung der klinischen Elektrophysiologie in Deutschland

**DOI:** 10.1007/s00399-024-01002-4

**Published:** 2024-02-29

**Authors:** Ludger Seipel, Fokko de Haan

**Affiliations:** 1 Tübingen, Deutschland; 2 Historisches Archiv, Deutsche Gesellschaft für Kardiologie – Herz- und Kreislaufforschung e. V, Grafenberger Allee 100, 40237 Düsseldorf, Deutschland

Nachdem die Arbeit von Scherlag et al. über die Ableitung von Aktionspotenzialen des His-Bündels beim Menschen Ende 1969 erschienen war, gab es in Deutschland kaum eine Reaktion. Lediglich M. Schlepper erkannte sofort die Bedeutung des Verfahrens, das er als Erster in Deutschland klinisch einsetzte. Er publizierte zusammen mit H. Neuss erste Ergebnisse der „His-Bündel-Elektrographie“ 1971. Wir in Düsseldorf wachten erst auf, als 1970 eine Arbeit über die klinische Bedeutung der Methode bei Patienten mit AV-Überleitungsstörungen erschien. Unglücklicherweise wollten wir wie Scherlag primär tierexperimentelle Erfahrung sammeln. So verstrich viel Zeit, indem wir versuchten, bei Hundeexperimenten, etwa in der Physiologie, vorher *schnell* ein His-Potenzial abzuleiten, was nie erfolgreich war. (Später benutzte H. Djonlagic dieses Modell zur Testung neuer Antiarrhythmika.) So wechselten wir in die Klinik, und bald wunderte sich der erste erfolgreiche Patient über unseren *Indianertanz* im Katheterraum. Eine weiterführende Arbeit war nur möglich durch die Bildung einer Arbeitsgruppe mit G. Breithardt und M. Borggrefe. In den folgenden Jahren entstanden mehrere elektrophysiologische Zentren, zum Teil mit Kollegen, die wie M. Runge oder K. Lang ihre *Lehrzeit* in den USA verbracht hatten.

Wissenschaftlich war nach der AV-Blockierungen der Sinusknoten („black box“) der nächste Arbeitsschwerpunkt. So wurde über die *richtige* Bestimmung der sinuatrialen Leitungszeit (SACT) heftig diskutiert. Die Grundlagen hierzu hatte G. Steinbeck aus der damaligen Arbeitsgruppe von B. Lüderitz in aufwendigen experimentellen Untersuchungen während eines Forschungsaufenthalts in Maastricht geschaffen. In den Sitzungen zu diesem Thema auf dem ESC-Kongress 1976 lernten wir auch einige Kollegen aus Ostdeutschland kennen: G.H. von Knorre, F.J. Rostock, H.J. Volkmann. Hier wurde die SN-Erholungszeit häufig mittels Ösophagusstimulation ermittelt, die von westdeutschen Patienten kaum toleriert wurde. Diese Ära endete mit der Erkenntnis, dass diese Parameter im Hinblick auf therapeutische Konsequenzen nicht besser waren als die Klinik und das Langzeit-EKG.

Dennoch stiegen die Patientenzahlen weiter an, da zunehmend tachykarde Rhythmusstörungen untersucht wurden. Mittels programmierter Stimulation wurde getestet, ob ein Antiarrhythmikum die Initiierung der Tachykardie verhindern konnte. Im positiven Fall wurde der Patient auf diese Substanz oral eingestellt. Falls es nicht gelang, ein effektives Medikament zu finden, wurden Patienten mit ventrikulären Tachykardien und mit gefährlichen Arrhythmien beim WPW-Syndrom einer elektrophysiologischen Operation zugeführt. Hierbei wurde am offenen Herzen das arrhythmogene Areal bzw. die akzessorische Bahn durch *Mapping* mittels handgehaltener Elektrode lokalisiert und chirurgisch ausgeschaltet, ein Verfahren, das von den Chirurgen (in Düsseldorf W. Bircks und J. Ostermeyer) viel Geduld erforderte. Diese Technik wurde insbesondere auch in Hannover durchgeführt (H. Klein, K.P. Bethge mit G. Frank). Sie war sehr effektiv, aber mit einer relativ hohen Operationsmortalität belastet.

Außerdem wurde mittels programmierter Stimulation untersucht, wie weit die Gefährdung von Patienten im Hinblick auf den plötzlichen Herztod vorausgesagt werden konnte. Die entsprechenden Studien zeigten allerdings, dass zwar eine gewisse Korrelation zwischen induzierten und spontanen Tachykardien bestand, nicht aber zum plötzlichen Herztod (G. Breithardt, B.D. Gonska).

Eine erste Alternative zur Operation war die Anfang der 1980er Jahre entwickelte DC-Ablation. Zunächst wurde hierbei der Stromstoß von einem externen Defibrillator über eine Plattenelektrode direkt auf den Elektrodenkatheter geleitet. Das Verfahren wurde primär dazu benutzt, um bei therapieresistenten Fällen von Vorhofflimmern mit schneller Überleitung das His-Bündel zu unterbrechen (T. Pop, Th. Meinertz). Bald erfolgten auch erste Versuche bei ventrikulären Tachykardien und akzessorischen Bahnen. Die Technik hatte aber den großen Nachteil einer erheblichen myokardialen Schädigung, so dass mehrfache Anwendungen, insbesondere bei kardialer Vorschädigung, kaum möglich waren.

Daher stellte die Einführung der HF-Ablationstechnik 1986 eine therapeutische Zeitenwende in der Elektrophysiologie dar. Der erste klinische Einsatz eines solchen Geräts erfolgte von G. Breithardt und M. Borggrefe zeitgleich mit einer Pariser Arbeitsgruppe. Unabhängig hiervon hat K.H. Kuck die gleiche Methode entwickelt. Jetzt stellten multiple Anwendungen kein Problem mehr dar. Allerdings war es gegenüber der DC-Technik sehr viel schwieriger, mit dem punktuellen Ablationsverfahren den Fokus bzw. Reentry-Kreis zu treffen, da es noch nicht die heutigen Mapping-Systeme gab. Ein besonderes Problem stellte die teilweise komplexe Anatomie akzessorischer AV-Verbindungen beim Präexzitationssyndromen dar (K.H. Kuck).

Es blieb nur noch eine Rhythmusstörung, die nicht mittels Kathetertechnik behandelt werden konnte: Vorhofflimmern. Bei Therapieversagen der Antiarrhythmika und schwerwiegender Symptomatik gab es nur die Operation am offenen Herzen (Maze-Operation). Erst in den 1990er Jahren führten die Arbeiten der Gruppe in Bordeaux zum Erfolg. Allerdings setzte sich das Verfahren in Deutschland nur relativ zögerlich durch, einmal wohl wegen der technischen Schwierigkeiten; es gab ja noch keine Ringkatheter etc. Zum anderen war das damalige Vorgehen mit potenziell tödlichen Komplikationen belastet.

Durch die weitere Entwicklung, insbesondere auch in technischer Hinsicht, wurde die klinische Elektrophysiologie zunehmend zu einer eigenen Disziplin im Rahmen der Kardiologie, was teilweise zur Ausgliederung selbstständiger Einheiten führte. Den heutigen Akteuren ist kaum noch vorstellbar, wie schwierig der Weg zur modernen klinischen Elektrophysiologie war. Dennoch ist es retrospektiv ein besonderes Glück gewesen, diese Pionierzeit miterlebt zu haben.

## Notes

The supplement containing this article is sponsored by Abbott, Biosense Webster, Biotronik, Boston Scientific, Deutsche Herzstiftung, Medtronic, MicroPort, OSYPKA and ZOLL.

